# Improving Lidocaine Topicalisation Practices in Paediatric Airway Management: A Quality Improvement Project

**DOI:** 10.7759/cureus.108676

**Published:** 2026-05-11

**Authors:** Christian Whiteley, Gareth Lodwick

**Affiliations:** 1 Faculty of Medicine, University of Southampton, Southampton, GBR; 2 Anaesthetics, University Hospital Southampton National Health Service (NHS) Foundation Trust, Southampton, GBR

**Keywords:** airway management, airway topicalisation, laryngospasm, lidocaine, lignocaine, paediatric airway, paediatric anaesthesia, quality improvement, topical anaesthesia, topicalisation

## Abstract

Background

Topical lidocaine is routinely used on paediatric vocal cords in surgeries requiring intubation or open airway procedures to suppress reflexes and coughing. Internationally and at University Hospital Southampton (UHS), a lack of standardised technique among anaesthetists has led to complications, including a case of toxicity, and confusion among recovery staff. This quality improvement (QI) project aimed to assess and improve practice against the recognised guidelines.

Methods

An anonymous questionnaire was distributed to consultant paediatric anaesthetists at UHS to document maximum lidocaine dosage (mg/kg), concentration (%), nil-by-mouth (NBM) time (minutes), administration device and respondents' reported incidents. The findings were then presented to respondents at an educational meeting, and guidelines were advocated to promote standardisation. Two weeks later, an identical follow-up questionnaire assessed changes in practice.

Results

Thirty-two participants were invited to participate in each survey. Surveys 1 and 2 received 22 (68.8%) and 15 (46.9%) responses, respectively. Median maximum dosages did not differ from the guidelines (4.0 mg/kg): 3.0 mg/kg (range 2.0-9.0 mg/kg, p=0.564) and 4.0 mg/kg (range 2.0-9.0 mg/kg, p=0.483). The distribution was unchanged across surveys (p=0.680), but adherence to guideline dosage increased from 2/22 (9.1%) to 8/15 (53.3%) (p=0.006). Median NBM time in Survey 1 was 120 minutes (range 60-360 minutes), significantly exceeding the guidelines (p<0.001), but decreased to 60 minutes (p=0.066, range 60-300 minutes), aligning with guidelines, and adherence improved from 8/22 (36.4%) to 11/15 (73.3%) (p=0.045). The choice of administration device was unchanged (p=0.850). No respondents reported using a lidocaine concentration above the recommended 4%. The 95% confidence intervals were calculated for the estimates.

Discussion

Substantial variation was identified in consultant anaesthetists’ practice regarding this technique, consistent with findings from national and international surveys. Following the intervention, the median dosage remained aligned with the recommended guidance of 4.0 mg/kg, but adherence increased significantly. In contrast, reported NBM times showed marked deviation from the recommended standard of 60 minutes, with excessive NBM times commonly being used. Post-intervention, both median NBM time and the proportion of respondents adhering to the standard improved substantially, although heterogeneity persisted. The choice of administration device was unchanged. This QI demonstrates that simple educational interventions can improve adherence to airway topicalisation standards and may inform similar local QI projects in other institutions where practice variability remains unidentified.

## Introduction

Topical lidocaine is commonly used to suppress protective airway reflexes, including the gag and laryngeal reflexes. Its indications therefore include facilitating open airway procedures (e.g. endoscopy, bronchoscopy), tracheal intubation and direct surgery on vocal cords [1,2].

Considerable variation exists in how anaesthetists use this technique, which several surveys throughout the UK have identified. These include a survey conducted by the Association of Paediatric Anaesthetists of Great Britain and Ireland (APAGBI) and a large international survey published in 2023 involving 1,501 respondents across 69 countries, which demonstrated heterogeneity in key aspects of practice [1,3]. These include maximum dosage, lidocaine concentration, administration device, and recommended nil-by-mouth (NBM) times following application. Notably, whilst most APAGBI respondents reported using 3.0 mg/kg, many reported doses of up to 10 mg/kg, in contrast to the international study in 2023 that reported a maximum dose of only 5.0 mg/kg [1,3].

This variability is likely driven by limited evidence and conflicting guidance. For example, the British National Formulary (BNF) for Children recommends a maximum dose of 3.0 mg/kg, whereas guidance from Alder Hey Children’s Hospital suggests a dose of up to 4.0 mg/kg [4,5]. Furthermore, whilst potentially toxic plasma lidocaine concentrations in adults are quoted at 10.0 μg/ml, no similar toxic concentrations have been published for paediatric patients [1,6]. Such discrepancies may contribute to uncertainty and variation in clinical practice.

Physiological factors may further compound this issue. The greater vascularity of paediatric vocal cords may increase lidocaine’s absorption rate, meaning the peak serum concentration is reached more quickly and is more pronounced than in adults [1,7]. In addition, systemic absorption of lidocaine varies in different sites of the upper aerodigestive tract, and thus site of application [8,9]. During topicalisation, small volumes may be removed by suction, some will be swallowed, and some may pass passively into the oesophagus, resulting in unpredictable systemic exposure [1,8,9]. By inference, this means multiple administration routes, combined with influence from adult practice, may increase the risk of lidocaine toxicity in paediatric patients [1,8,10]. With respect to post-procedural NBM time, the APAGBI survey reported that while 90% of anaesthetists impose a NBM period, durations ranged widely from 20 to 240 minutes. This variation may reflect the limited paediatric-specific evidence regarding safe re-feeding times as there is uncertainty regarding the duration of inhibition of protective reflexes [1].

Despite the recognition of practice variation, no published quality improvement (QI) initiatives have sought to address this issue through intervention and re-audit. At University Hospital Southampton (UHS), inconsistent practice has resulted in confusion among recovery staff regarding re-feeding times and has been associated with one reported case of lidocaine toxicity.

This QI project, therefore, has the following objectives:

Primary aim: To assess the variation in lidocaine topicalisation practices at UHS and implement a targeted educational intervention to improve adherence to evidence-based guidelines, followed by re-evaluation of practice. Key aspects of practice examined included: dosage, local anaesthetic concentrations, NBM times and administration devices. The intervention was designed to reduce the risk of future local anaesthetic toxicity, minimise uncertainty regarding re-feeding in recovery and provide a model for identifying and addressing similar practice variation in other institutions.

Secondary aim: To explore self-reported cases of incidents among anaesthetists, and their association with the factors outlined above.

The preliminary results from this study were presented as an ePoster and published in an abstract at the Resident Doctors Conference, hosted by the Association of Anaesthetists, June 26, 2025.

## Materials and methods

Study design and context

The aspects of practice evaluated included the use of airway topicalisation, local anaesthetic agent and concentration (%), maximum lidocaine dose used (mg/kg), administration device, patient age groups and clinical contexts, NBM time (minutes) and reported incidents. Reported incidents were captured as free-text qualitative responses and reflected any complications or concerns identified by respondents, rather than predefined outcome measures. Examples included critical life-threatening events or episodes of aspiration or difficulty swallowing.

These variables were selected due to their identified national and international heterogeneity and their clinical relevance for patient safety [1]. The Standards for Quality Improvement Reporting Excellence, version 2.0 (SQUIRE 2.0) guidance was used to assist in the design of this QI project [11].

Participants

Eligible participants for the survey were consultant paediatric anaesthetists employed at UHS. Associate specialists who had significant independent practice were also included. Respondents who reported routinely using topical lidocaine for paediatric airway management were included in the statistical analysis. Those who reported not using the technique were excluded.

No locum consultants were in post at UHS during the audit period. Trainees, fellows and specialty doctors were excluded to ensure that responses reflected established clinical practice and local decision-making responsibility.

Data collection

Data were collected using an anonymous electronic questionnaire distributed via departmental email on September 10, 2024. The survey included multiple-choice and free-text questions to capture precise values for dose and NBM time. Responses were collected before and after the educational intervention, two weeks apart. The questionnaire was designed using Qualtrics (Qualtrics XM, Provo, UT, USA) and is provided in the Appendix.

Intervention

The intervention consisted of a structured in-person teaching session delivered to consultant paediatric anaesthetists at UHS, incorporated into routine mandatory departmental teaching. The session, hosted by a consultant paediatric anaesthetist and the first author, lasted approximately 45 minutes and was supported by a formal PowerPoint presentation. The content included: (1) a review of the literature underpinning safe use of topical lidocaine on paediatric airways, (2) discussion of previously encountered complications, including lidocaine toxicity and uncertainty regarding re-feeding times in recovery and (3) advocation of evidence-based guidelines for practice. The guidelines used are outlined below. The session incorporated an interactive component, allowing attendees to ask questions and clarify areas of uncertainty.

Further details on the PowerPoint presentation used to deliver the intervention are provided in the Appendix to facilitate reproducibility.

Lidocaine Dosage

A maximum dose of 4.0 mg/kg was advocated with a concentration ≤4%. This recommendation was based on a prospective study conducted in Melbourne, Australia, which evaluated plasma lidocaine concentrations following topical airway administration of 4% lidocaine, 4.0 mg/kg, in 96 children aged two weeks to 12 years. Although occasional plasma concentrations exceeded traditionally accepted thresholds of 8.0 μg/ml in all ages to 13 children, no clinical toxicity was observed [8]. APAGBI guidance subsequently supported this dose, stating that it would be wise to avoid higher doses, particularly in neonates, young infants or where increased absorption may occur [1].

Nil-by-Mouth Time

A 60-minute NBM period following airway topicalisation was advocated. While the precise degree of laryngeal reflex recovery required to facilitate a safe swallow is unknown, it is wise to assume aspiration is still a risk for some time after the return of the first response [1]. Evidence from in-office laryngeal procedures supports withholding oral intake for 30-60 minutes [10]. A 60-minute period was therefore considered a pragmatic balance between the safe recovery of laryngeal reflexes and causing minimal distress to the child [9,12,13].

This recommendation was further supported by APAGBI survey data, indicating that 10% of respondents did not withhold oral intake at all, and to this date, no reported cases of aspiration have occurred in adult or paediatric literature [1].

Administration Device

Use of the mucosal atomisation device (MADgic®, Teleflex Incorporated, Wayne, PA, USA; MAD henceforth) was advocated as standard practice. Metered-dose sprays typically deliver much larger doses, with around 10 mg per spray and often require multiple sprays, reaching up to 30-40 mg and risking excessive dosing in lighter patients (<10 kg) [14,15].

Although Jelco® devices (IMS Euro Ltd, UK) are widely used, reports from nearby institutions have described detachment of the tip into the airway. The MAD delivers a fine mist, which is less irritant and better tolerated by the airway. Furthermore, it is more likely to reach the peripheral airway and delivers over a wider area [1].

Re-audit

An identical questionnaire was distributed on 18 October 2024, following the educational intervention, to assess whether practice had changed towards the advocated standards.

Measures and outcomes

The primary outcomes were changes in: Reported maximum lidocaine dose, reported NBM time, administration device and the proportion of respondents adhering to the advocated standards.

Secondary outcomes included: Reported incidents and their association with the primary outcomes.

Statistical analysis

Responses were treated as independent samples and SPSS, version 29.0 (IBM Corp, Armonk, NY) was used for data analysis. Continuous data were summarised as medians. Wilcoxon signed-rank tests were used as one-sample non-parametric tests to compare reported medians against the advocated standards. Differences between surveys were analysed with Mann-Whitney U tests. Categorical variables were compared using χ² or Fisher’s exact tests. 95% confidence intervals were calculated for key estimates. Outliers were predefined for maximum dosage as ≥6.0 mg/kg or ≤2.0 mg/kg, and NBM times <60 or >120 minutes and these were reviewed individually on a case-by-case basis.

Ethical considerations

Ethical approval was obtained from the Ethics and Research Governance Committee (ERGO) (reference number: 92015). The application detailed the QI rationale, methodology, and risk assessment. Participants provided informed consent via the first question of the questionnaire. No identifiable data were collected, and participation was voluntary.

## Results

Participants and data structure

A total of 32 consultant paediatric anaesthetists were invited to participate. Survey 1 and Survey 2 received 22 (68.8%) and 15 (46.9%) valid responses, respectively. One respondent in Survey 1 reported not using this technique and was excluded; all remaining responses were included in the analysis.

Responses were anonymous and unpaired, as individual respondents could not be matched between surveys. No respondents changed institutions between surveys. The guideline standards used as the basis for audit are detailed in the Methods section.

Maximum lidocaine dose

**Figure 1:**
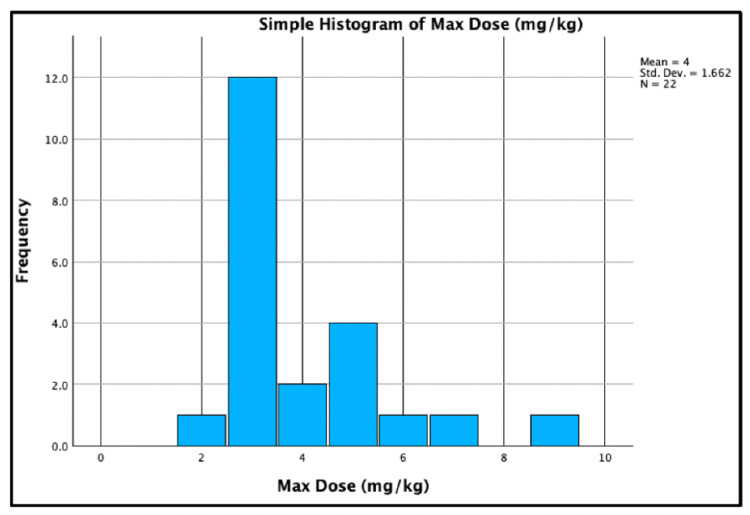
Histogram showing distribution of maximum doses (mg/kg) from Survey 1

**Figure 2:**
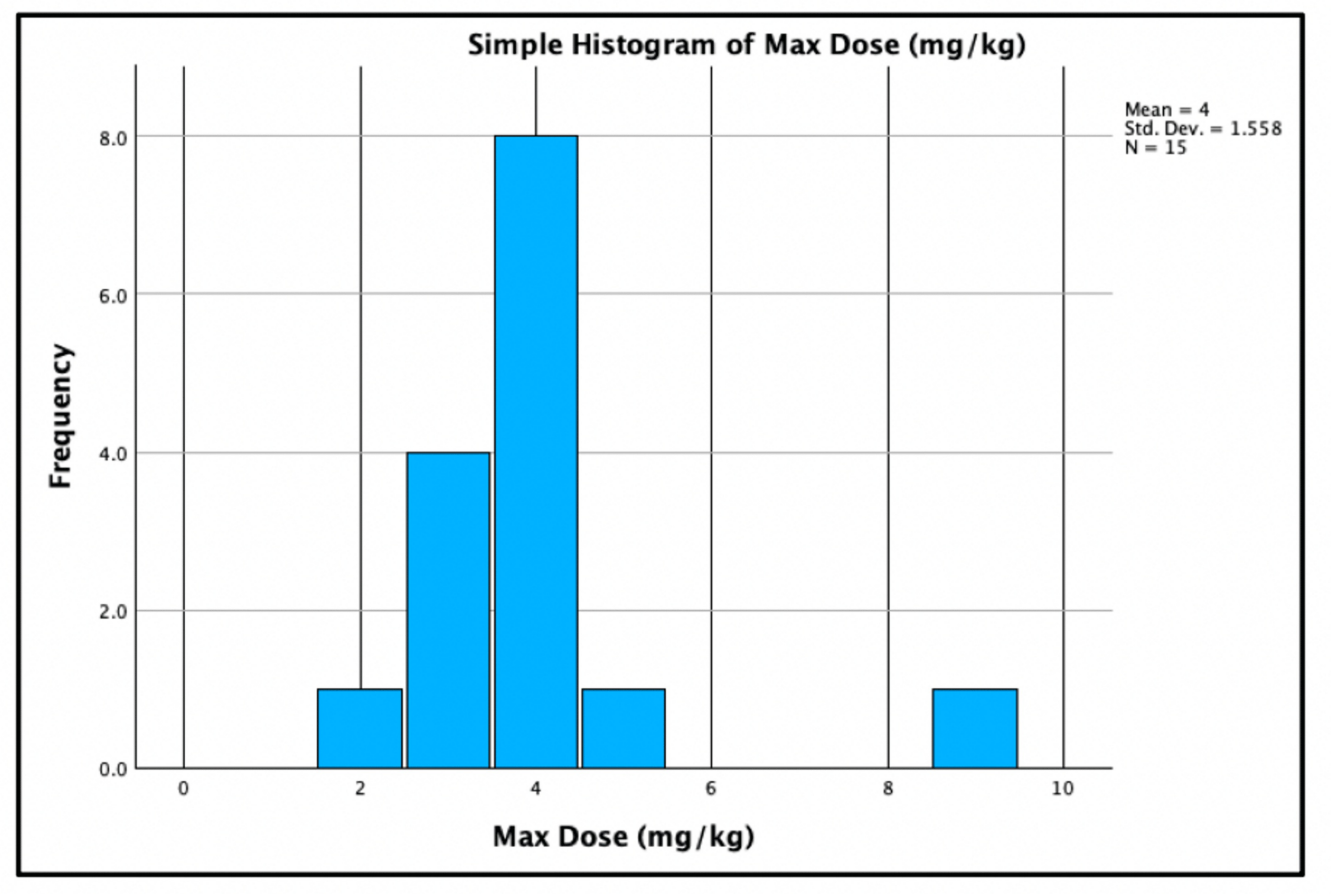
Histogram showing distribution of maximum doses (mg/kg) from Survey 2

The reported maximum lidocaine doses were non-normally distributed; therefore, medians and ranges are presented. The median reported maximum dose was 3.0 mg/kg (range 2.0-9.0 mg/kg) in Survey 1 and 4.0 mg/kg (range 2.0-9.0 mg/kg) in Survey 2. Figures 1, 2 summarise the findings.

When compared with the recommended standard of 4.0 mg/kg, neither survey median differed significantly (Wilcoxon signed-rank test: Survey 1, W=90.50, p=0.564; Survey 2, W=10.00, p=0.483). A comparison of dose distributions between surveys showed no statistically significant change following the intervention (Mann-Whitney U test: U=179, p=0.680).

However, the proportion of respondents reporting use of the recommended maximum dose of 4.0 mg/kg increased significantly from 2/22 (9.1%) in Survey 1 to 8/15 (53.3%) in Survey 2, representing an absolute increase of 44.2% (95% confidence interval (CI) 14.4% to 67.1%; Fisher’s exact test, p=0.006). Table 1 summarises the statistical test outcomes.

**Table 1:** Statistical test outcomes for maximum dosage (mg/kg) for Surveys 1 and 2

	Survey 1	Survey 2	Survey comparisons
Range (mg/kg)	7.0	7.0	-
Median (mg/kg)	3.0	4.0	-
Wilcoxon signed-rank comparing to advocated 4.0mg/kg	P=0.564 (no difference)	P=0.483 (no difference)	-
Mann-Whitney U p-value comparing survey distributions	-	-	P=0.680 (no change)
Fisher's exact p-value comparing proportions between surveys	-	-	P=0.006 (significant change)

Despite this improvement, marked heterogeneity persisted, with reported doses ranging from 2.0 mg/kg to 9.0 mg/kg in both surveys.

No consistent association was identified between predefined dose outliers (≤2.0 mg/kg or ≥6.0 mg/kg) and reported incidents (Table 2).

**Table 2:** Individual cases of ‘outliers’ in Surveys 1 and 2 classed by a maximum dosage of ≤2.0 mg/kg or ≥6.0 mg/kg NBM: Nil-by-mouth; ENT-LTB: ENT-laryngo-tracheo-bronchoscopy; OGD-Scope: Oesophago-Gastro-Duodenoscopy.

	Max dose (≤2.0mg/kg, ≥6.0mg/kg)	Survey Number	NBM time (minutes)	Administration device	Age Groups	Patient Groups	Anaesthetic	Critical incidents	Incidents aspiration/diff swallowing
Outlier 1	2.0	1	240	Jelco	Infants Children Teenagers	ENT-LTB	Lidocaine 1%	None recorded	None recorded
Outlier 2	9.0	1	60	Jelco	Infants Children Teenagers	ENT-LTB ENT-Tonsillectomy	Lidocaine 1% Lidocaine 2%	None recorded	None recorded
Outlier 3	7.0	1	120	Jelco and MAD	Neonates Infants Children Teenagers	ENT-LTB	Lidocaine 1% Lidocaine 2%	None recorded	None recorded
Outlier 4	6.0	1	120	Jelco and MAD	Neonates Infants Children Teenagers	ENT-LTB OGD-Scopes Bronchoscopy	Lidocaine 1%	Laryngospasm	None recorded
Outlier 5	2.0	2	240	Jelco	Neonates Infants Children	ENT-LTB	Lidocaine 1%	None recorded	None recorded
Outlier 6	9.0	2	180	Jelco	Children	ENT-LTB	Lidocaine 2%	None recorded	None recorded

Nil-by-mouth (NBM) time

**Figure 3:**
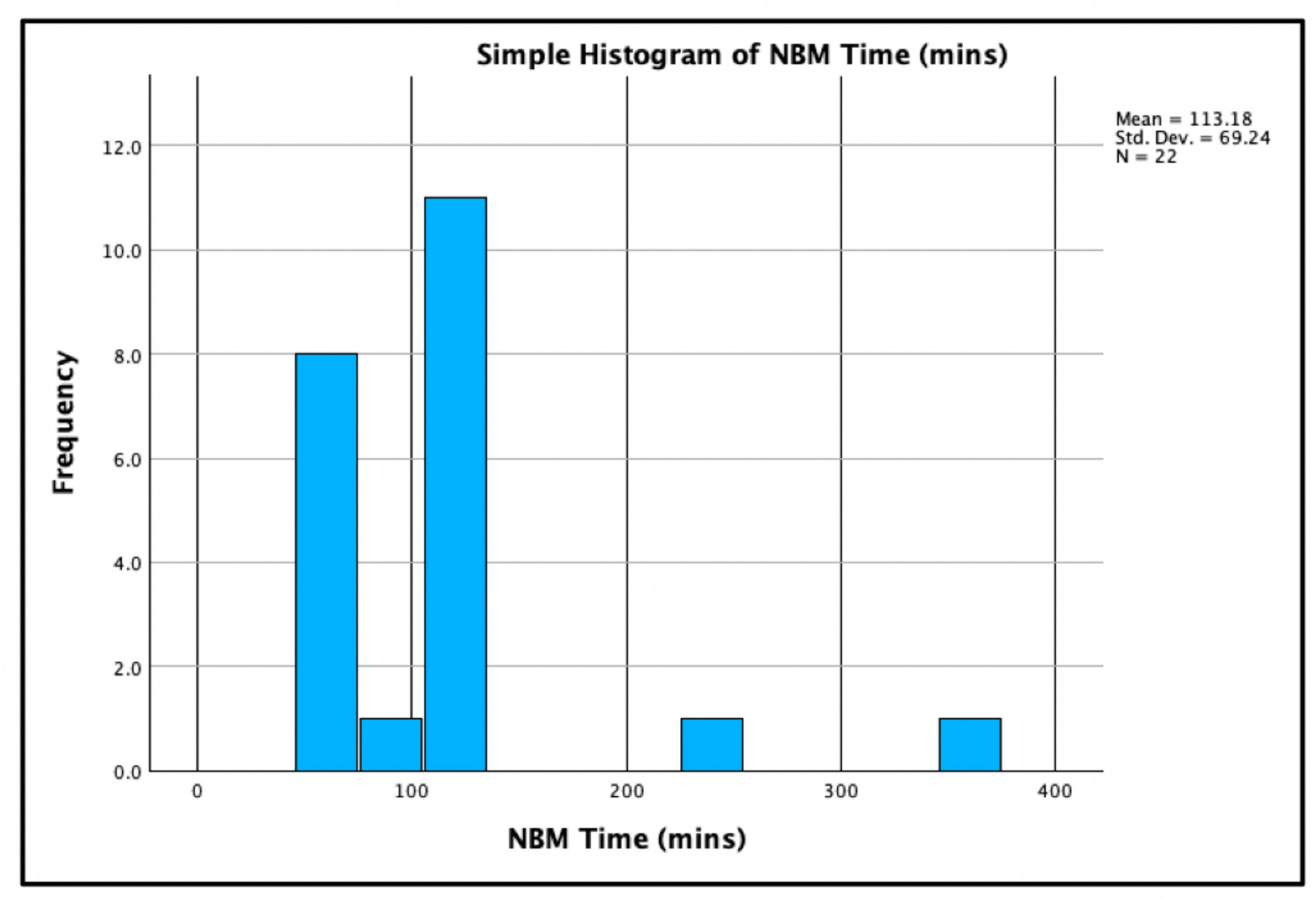
Histogram showing distribution of nil-by-mouth (NBM) time (minutes) from Survey 1

**Figure 4:**
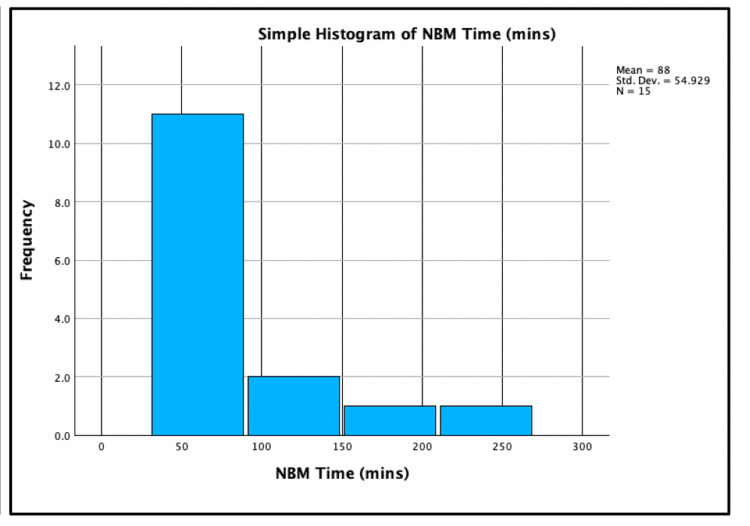
Histogram showing distribution of nil-by-mouth (NBM) (minutes) from Survey 2

NBM times were non-normally distributed. In Survey 1, the median NBM time was 120 minutes (range, 60-360 minutes), which differed significantly from the recommended standard of 60 minutes (Wilcoxon signed-rank test: W=105, p<0.001). In Survey 2, the median NBM time reduced to 60 minutes (range, 60-300 minutes), which no longer differed significantly from the standard (Wilcoxon signed-rank test: W=10, p=0.066). Figures 3, 4 summarise the findings.

A comparison of NBM time distributions between surveys did not demonstrate a statistically significant difference (Mann-Whitney U test: U=113.5, p=0.112). However, the proportion of respondents adhering to the standard increased significantly, from 8/22 (36.4%) in Survey 1 to 11/15 (73.3%) in Survey 2. This represents an absolute increase of 36.9% (95% CI: 4.3% to 59.9%; χ²=4.88, p=0.045). Table 3 summarises the statistical test outcomes.

**Table 3:** Statistical test outcomes for NBM time (minutes) in Surveys 1 and 2 NBM: Nil-by-mouth.

	Survey 1	Survey 2	Survey comparisons
Range (minutes)	300	240	-
Median (minutes)	120	60	-
Wilcoxon signed-rank comparing to advocated 60 minutes	P<0.001 (significant difference)	P=0.066 (no difference)	-
Mann-Whitney U p-value comparing survey distributions	-	-	P=0.112 (no change)
Chi-square p-value comparing proportions between surveys	-	-	P=0.045 (significant change)

From Survey 1 to Survey 2, the mode was reduced from 120 minutes to 60 minutes. The range reduced slightly from 60-360 minutes to 60-300 minutes, showing that the variation persisted. One respondent said they generally allow sips of water immediately after topicalisation and no critical incidents were recorded with this respondent. 

No reliable association was identified between predefined NBM outliers (<60 minutes or >120 minutes) and reported incidents (Table 4).

**Table 4:** Individual cases of ‘outliers’ in Surveys 1 and 2 classed by a NBM time of <60 minutes or >120 minutes NBM: Nil-by-mouth; ENT-LTB: ENT-laryngo-tracheo-bronchoscopy.

	NBM time (>120 minutes)	Survey Number	Max dose (mg/kg)	Administration device	Age Groups	Patient Groups	Anaesthetic	Critical Incidents	Incidents aspiration/diff swallowing	Comments
Outlier 1	360	1	3.0	Jelco and MAD	Neonates Infants Children Teenagers	ENT-LTB ENT-Tonsillectomy	Lidocaine 1%	None recorded	None recorded	NBM for fluids till sending
Outlier 2	240	1	2.0	Jelco	Infants Children Teenagers	ENT-LTB	Lidocaine 1%	None recorded	None recorded	
Outlier 3	240	2	2.0	Jelco	Neonates Infants Children	ENT-LTB	Lidocaine 1%	None recorded	None recorded	
Outlier 4	180	2	9.0	Jelco	Children	ENT-LTB	Lidocaine 2%	None recorded	None recorded	

Device used for administration

The use of a Jelco® device decreased marginally from 18/22 (81.8%) in Survey 1 to 12/15 (80.0%) in Survey 2 (absolute change −1.8%; 95% CI: −22.3% to 29.3%). This difference was not statistically significant (Fisher’s exact test, p=1.000).

The use of the MAD® mucosal atomisation device increased slightly from 14/22 (63.6%) to 10/15 (66.7%) (absolute change +3.1%; 95% CI: −30.6% to 27.0%), which was not statistically significant (χ²=0.036, p=0.850).

**Figure 5:**
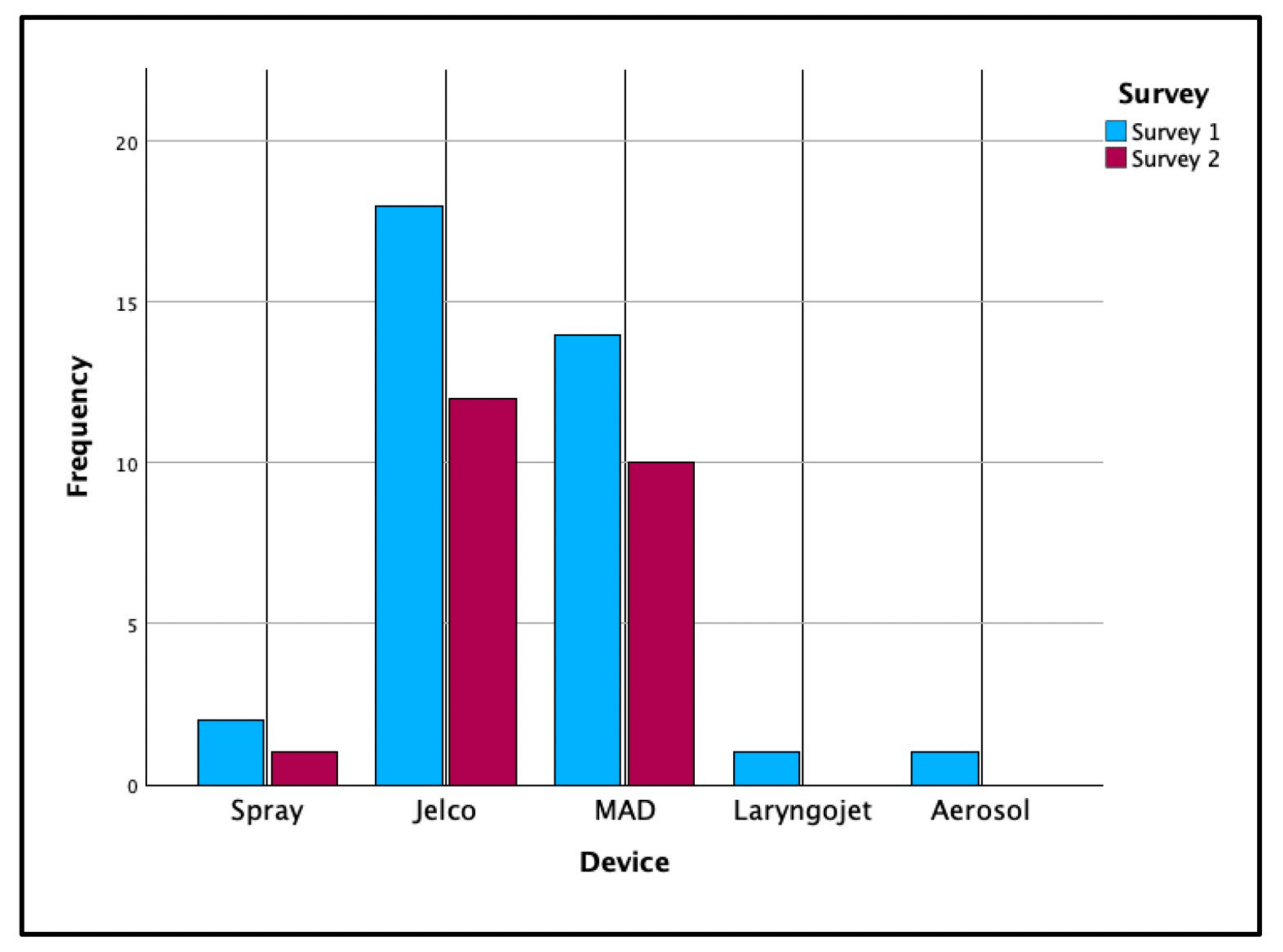
Clustered bar graph of showing distribution of devices used to administer in Surveys 1 and 2 Jelco, IMS Euro Ltd, UK; MADgic: Teleflex Inc., Wayne, PA, USA; Laryngojet, Teleflex Incorporated, Wayne, PA, USA

Throughout both surveys, six respondents stated that the device depends on the patient’s age/weight. Four of these explained that they use the Jelco® device in smaller patients and the MAD® device in older children. Due to low frequencies, statistical analysis of other devices was not undertaken. Figure 5 summarises the findings.

Lidocaine concentration used

**Figure 6:**
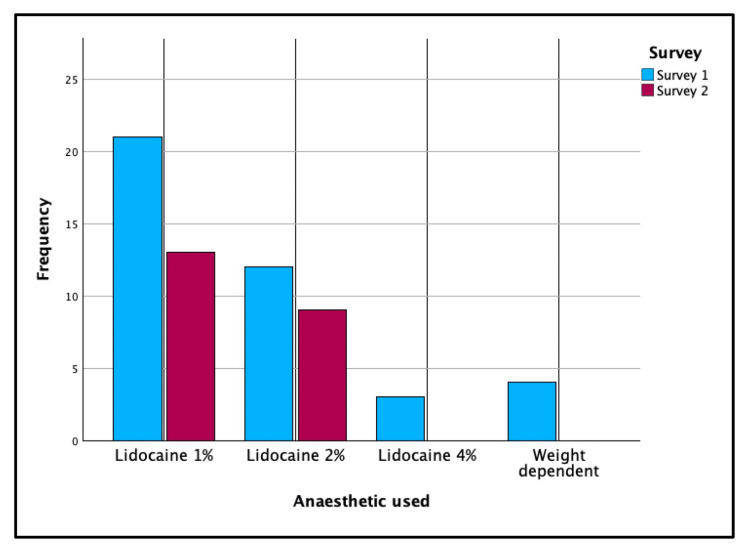
Clustered bar graph showing distribution of anaesthetic used in Surveys 1 and 2 Variables with zero responses were omitted from graphical presentation for clarity.

All respondents use only 1% or 2% lidocaine, except three respondents who reported using 4% in Survey 1. Two of these stated that they use 1% for babies/neonates and increase the concentration up to 4% for teenagers (weight-dependent). No respondents reported routine use of concentrations greater than 4%. Figure 6 summarises the findings.

Reported incidents

In Survey 2, one case of difficulty swallowing occurred when nasal cocaine was used before a throat pack, the same respondent who reported the non-critical laryngospasm. No such cases were recorded in Survey 1.

Two of the three cases of laryngospasm were reported when 4% lidocaine was used. Both respondents stated that this concentration was reserved for teenage patients. One additional incident was reported following use of a metered-dose spray, although further details were not provided.

**Figure 7:**
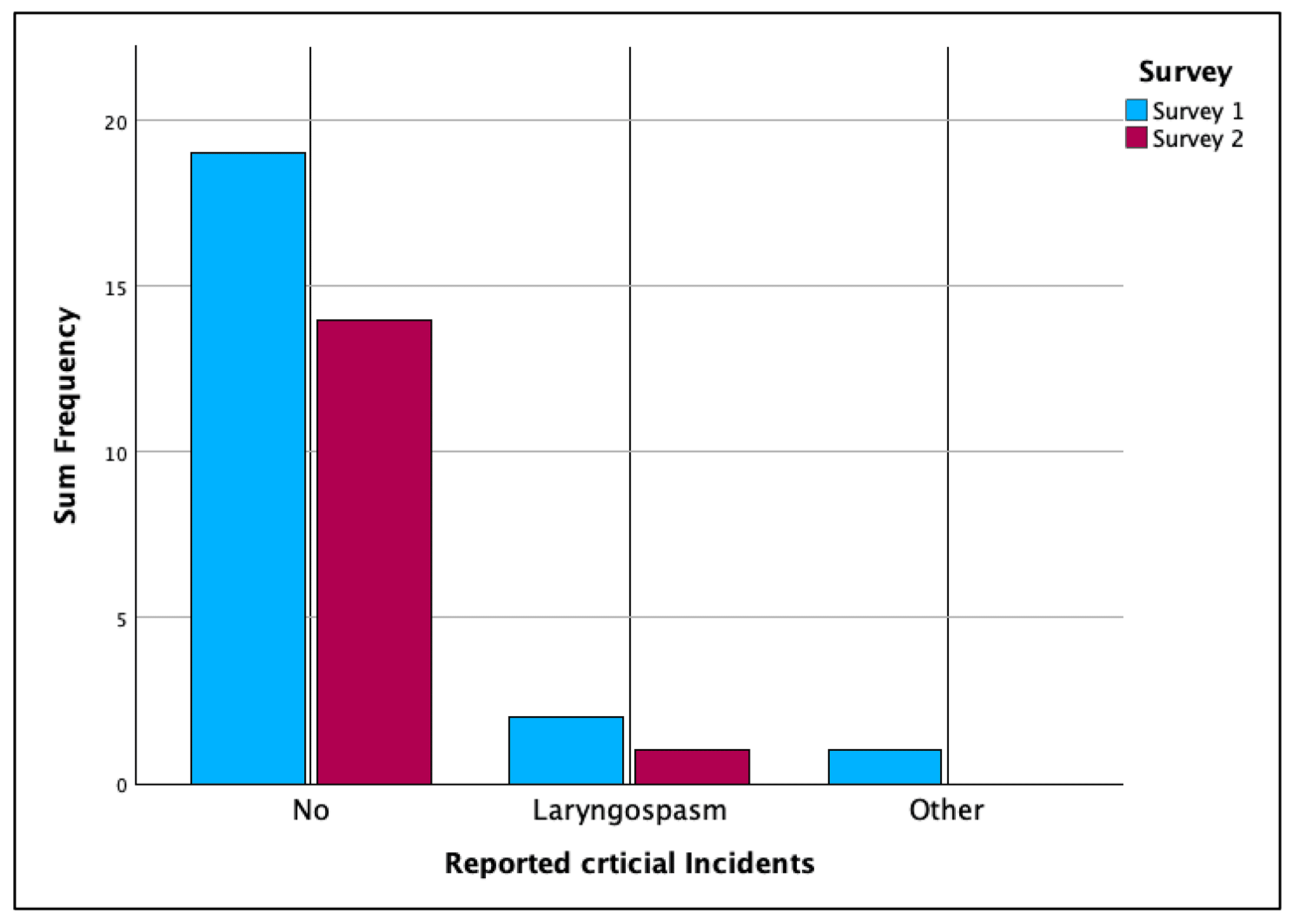
Clustered bar graph of reported critical incidents in Surveys 1 and 2. ‘Other’ refers to an incident when the metered-dose spray was used.

Due to low event frequency, formal statistical analysis of incident data was not undertaken and no consistent relationship was identified between reported incidents with maximum dosage, NBM time, administration device, or lidocaine concentration. Figure 7 and Table 5 summarise the findings.

**Table 5:** Individual cases of incidents in Surveys 1 and 2. Incidents are classed as either ‘critical incident’ or ‘incident of aspiration or difficulty swallowing'. ENT-LTB: ENT-laryngo-tracheo-bronchoscopy. Laryngojet, Teleflex Incorporated, Wayne, PA, USA.

	Incident	Survey Number	Max Dose (mg/kg)	NBM Time (minutes)	Administration Device	Age Groups	Patient Groups	Anaesthetic	Comments
Outlier 1	Laryngospasm	1	3.0	120	Jelco and MAD	Neonates, Infants, Children, Teenagers	ENT-LTB Bronchoscopy	Lidocaine 1% Lidocaine 2% Lidocaine 4%	1% neonates/infants, 4% teenagers, 2% for other age groups. Laryngospasm resolved after LA effect
Outlier 2	Laryngospasm	1	5.0	120	Jelco and Laryngojet	Neonates, Infants, Children, Teenagers	ENT-LTB Bronchoscopy	Lidocaine 1% Lidocaine 2% Lidocaine 4%	1% babies to 4% for teenagers Laryngojet if older Laryngospasm occurred due to insufficient depth of LA
Outlier 3	Incident when spray used	1	3.0	120	Jelco, MAD and Spray	Infants, Children, Teenagers	ENT-LTB	Lidocaine 1%	No more detail provided in respondent's answer
Outlier 4	Laryngospasm	2	3.0	120	Jelco	Neonates, Infants, Children, Teenage	ENT-LTB Bronchoscopy	Lidocaine 1%	MADgic if old enough
Outlier 5	Difficulty swallowing	2	3.0	60	Jelco and MAD	Neonates, Infants, Children, Teenage	ENT-LTB	Lidocaine 2%	Difficulty swallowing when nasal cocaine used prior to throat pack

## Discussion

Maximum lidocaine dosage

The median reported maximum lidocaine dose in both surveys was consistent with guidance advocated by the Melbourne study, APAGBI's review, and guidance from Alder Hey Children’s Hospital [1,4,8]. This suggests there was originally little deviation from guidelines. However, the wide range of reported doses (2.0-9.0 mg/kg) in both surveys indicates substantial inter-individual variation. This finding is consistent with other surveys, which also showed heterogeneity on a wider population scale, including 297 participants in APAGBI’s 2013 survey, which reported the same median with a maximum of 10.0 mg/kg, and the international study in 2023 showing a maximum of 5.0 mg/kg [1,3]. Importantly, the educational intervention resulted in a significant increase in the proportion of respondents using 4.0 mg/kg. This suggests that similar local QI projects can improve adherence to agreed standards, even when overall practice appears acceptable. Nevertheless, doses exceeding 4.0 mg/kg were still reported following the intervention, highlighting ongoing opportunities for further quality improvement.

Nil-by-mouth time

In contrast to the maximum dosage, Survey 1 demonstrated a marked deviation from the recommended NBM time, with a median of 120 minutes. This mirrors findings from APAGBI's survey but contrasts with evidence from in-office laryngeal procedures advocating shorter fasting periods [1,10].

Following the intervention, both the median NBM time and the proportion of respondents adhering to the recommended 60 minutes increased significantly. A reduced NBM time can limit unwarranted patient discomfort post-operatively, and the promoted standardisation could reduce future complications. However, heterogeneity persisted, with NBM times exceeding 60 minutes still reported in Survey 2. These findings present opportunities for future surveys and QI projects where varied practices on NBM time may be unidentified, and heterogeneity can be further reduced.

From a systems perspective, reducing variability in NBM times may also reduce uncertainty among operating department practitioners (ODPs) and recovery staff regarding safe re-feeding, addressing a key local concern that motivated this QI project.

Administration device

Survey 1 also demonstrated initial varied use of administration devices for this technique. Between surveys, this practice did not significantly change, with most participants still using Jelco® over the MAD® device and at least one participant using the spray in both surveys.

Several factors may explain this finding. First, multiple respondents indicated that device choice was influenced by patient size or age, suggesting that individual clinical judgement plays a substantial role. Secondly, established device preference and familiarity may make anaesthetists reluctant to change. Such context-dependent decision-making may mean that an education meeting alone may not be sufficient to shift this practice.

Nevertheless, this QI project provides valuable insight into device preferences, an area that is underrepresented. Future QI initiatives aiming to change device choice may require more intensive or targeted interventions, including exploration of rationale and potential practical barriers.

Incidents and adverse events

Reported incidents were rare across both surveys, and no consistent association was identified between adverse events and the variables assessed. The reliance on retrospective self-reporting and the short interval time between surveys may have contributed to this result; however, these results were anticipated, as this project was not designed to detect correlation with uncommon clinical events. Existing literature also reports that serious complications related to this technique are rare [1]. Most reported events of laryngospasm were attributed to inadequate depth of anaesthesia, which also aligned with APAGBI's findings [1]. Furthermore, the absence of reported aspiration events is consistent with previous paediatric studies, particularly given that no NBM times shorter than 60 minutes were recorded [16-18]. Notably, no incidents occurred in the participant who allowed sips of water immediately after topicalisation.

Limitations

As a single-centre, survey-based QI project, this study has limitations. First, it has a relatively small sample size compared to other surveys, limiting statistical power. However, given that 32 consultant paediatric anaesthetists practice at UHS, this sample size appropriately represents the local population.

Secondly, Survey 2 received seven fewer respondents, making comparative conclusions between the surveys less reliable despite attempts to improve response rates. The anonymous nature of the questionnaires prevented paired response testing and changes in practice for individuals could not be tracked. The self-reported nature and participant's awareness of being audited may also have caused inaccuracy in reflecting true clinical behaviour, as recall bias, social desirability bias or the Hawthorne effect may have led to potential overestimation of guideline adherence. While anonymity encouraged honest reporting, future studies could consider the use of unique identifiers to enable paired analysis, balanced against confidentiality concerns.

The QI project represents local culture at a single tertiary paediatric anaesthesia centre. Institutional norms and established individual practice patterns may, therefore, limit generalisability. In addition, although the intervention was delivered as intended with all consultant paediatric anaesthetists invited to the educational session, uptake of recommendations may have been influenced by factors such as seniority.

This QI project's primary aim was not to find an association between practice variables and incidents, and it was not powered to do so. Incident reporting was limited by the lack of a defined timeframe in survey questions, making it impossible to determine whether the event being described was a new or historical one. The short interval between surveys further limited the ability to detect changes in complication rates. Future QI projects should adopt a longer follow-up period between survey cycles, and prospective data collection would be required to assess the sustained impact of practice change on clinical outcomes.

## Conclusions

This QI project identified substantial variation in practice regarding this technique. The educational intervention successfully improved adherence to recommended maximum dosing and NBM time but did not significantly influence the choice of administration device. Despite improved alignment with guidelines, some practice heterogeneity persisted.

The QI project was not designed or powered to detect changes in rare adverse events, and no clear association was observed with the variables assessed. Nevertheless, improved standardisation of NBM times may reduce unnecessary patient discomfort and re-feeding uncertainty for recovery and ODP staff. These findings support the value of structured local QI projects in identifying and addressing unwarranted practice variation. They can be used to support other trusts implementing similar QI projects where this may be unidentified. Future work should explore alternative or more sustained interventions to further standardise practice and assess the long-term sustainability of change.

## Appendices

Appendix A: Consultant questionnaire used for data collection

**Table 6:** Questionnaire distributed to participants. The same questionnaire was used for both survey cycles.

Question no.	Question	Response type	Response options
1	Consent. Information sheet given detailing why this audit is taking place and why participant was selected. Then participant asked 'Can you confirm that you are over the age of 18, understand the information above and consent to taking part in this questionnaire?'	Multiple choice	Yes / No
2	Do you ever use topical local anaesthetic on paediatric patients’ vocal cords?	Multiple choice	Yes / No
3	What patient groups do you use this for?	Multiple choice (select all that apply)	ENT-LTB / ENT-tonsillectomy / General Surgery-Urology / OGD Scopes / Orthopaedic / Other (please state)
4	Which local anaesthetic do you use?	Multiple choice (select all that apply)	Lidocaine 1% / Lidocaine 2% / Lidocaine 4% / Lidocaine 10% / Levo/bupivacaine 2.5% / Levo/bupivacaine 5% / Other (please state)
5	What do you use as the maximum dose of lidocaine topically in mg/kg in paediatric patients?	Free text	-
6	What device do you use to administer?	Multiple choice (select all that apply)	Spray supplied with local anaesthetic / Jelco® / MADgic® device / Other (please state)
7	Which age groups do you use this technique for?	Multiple choice (select all that apply)	Neonates / Infants / Children / Teenagers
8	How long do you keep patients NBM (Nil-by-mouth) for?	Free text	-
9	Have you ever experienced a critical incident in theatre using this technique? Can you describe this incident?	Free text	-
10	Have you been made aware of any of your patients having difficulty with swallowing or aspiration in recovery? Can you describe this incident?	Free text	-

Appendix B: Definitions used in analysis 

Incidents are classed as either ‘critical incident (Q9)’ or ‘incident of aspiration or difficulty swallowing (Q10).’

Maximum lidocaine dosage outliers were classed as ≤2.0 mg/kg or ≥6.0 mg/kg.

NBM times that were classed as outliers were <60 min or >120 min.

Appendix C: Educational intervention PowerPoint presentation 

Slide 1 titled: *'Why is this clinical audit taking place.'* Detailed previous complications, including a case of lidocaine toxicity and confusion regarding re-feeding amongst recovery staff. Also highlighted the lack of current evidence for standardised guidelines in the literature.

Slides 2 and 3 titled: *'Maximum dose variation.'* Presented the results from Survey 1 regarding dosages and promoted the 4.0 mg/kg guideline using current evidence in the literature.

Slides 4 and 5 titled: *'NBM time variation.'* Presented the results from Survey 1 regarding NBM times and promoted the 60-min guideline using current evidence in the literature.

Slide 6 titled: '*Device used to administer.'* Presented the results from Survey 1 regarding administration devices and promoted the MAD device using current evidence in the literature.

Slide 7 titled: *'Local anaesthetic used.'* Presented the results from Survey 1 regarding local anaesthetic drugs and concentrations and promoted 1%-4% lidocaine concentration using current evidence in the literature.

Slides 8 and 9 titled: *'Incidents.'* Presented the results from Survey 1 regarding any observed cases of laryngospasm or incidents whilst using the procedure.

Slide 10 titled: *'Why this varied practice may have occurred.'* Used current literature to inform why variation is present at a local and international level.

Slide 11 titled: *'How to prevent this in the future.'* Summarised all guidelines that were introduced and promoted throughout the presentation, including maximum dosage, NBM time, administration device, preferred local anaesthetic and concentration, and advice on the onset/offset time of lidocaine.

Slide 12: References 
